# An exceptional perianal basal cell carcinoma with follicular differentiation mimicking Hemorrhoids: a rare case report

**DOI:** 10.1093/omcr/omag015

**Published:** 2026-03-23

**Authors:** Imane Boujguenna, Mohamed Essaid Ramraoui, Hind Boujguenna, Soufiane Abdouh, Abdelwahab Dallouri, Wissal Biad, Rawaa Bouaachra, Olaya Iaich, Wiam Sbai, Imane E L Ghanter, Fatima Boukis, Mohamed Amine Haouane, Ahmed Elguazzar

**Affiliations:** Guelmim Faculty of Medicine and Pharmacy, Ibn Zohr Agadir University Guelmim 81000, Morocco; Moulay Elhassan Military Hospital, Department of General Surgery, Guelmim 81000, Morocco; Private University of Marrakesh, Marrakesh 40000, Morocco; Private Practice Physician, Marrakesh 40000, Morocco; Guelmim Faculty of Medicine and Pharmacy, Ibn Zohr Agadir University Guelmim 81000, Morocco; Guelmim Faculty of Medicine and Pharmacy, Ibn Zohr Agadir University Guelmim 81000, Morocco; Guelmim Faculty of Medicine and Pharmacy, Ibn Zohr Agadir University Guelmim 81000, Morocco; Guelmim Faculty of Medicine and Pharmacy, Ibn Zohr Agadir University Guelmim 81000, Morocco; Guelmim Faculty of Medicine and Pharmacy, Ibn Zohr Agadir University Guelmim 81000, Morocco; Guelmim Faculty of Medicine and Pharmacy, Ibn Zohr Agadir University Guelmim 81000, Morocco; Al AMAL Pathological Anatomy Laboratory, Guelmim 81000, Morocco; Department of Pathology, Caddi Ayyad University of Marrakesh, Ibn Sina Military Hospital, Marrakesh 40000, Morocco; Moulay Elhassan Military Hospital, Department of General Surgery, Guelmim 81000, Morocco

**Keywords:** basal cell carcinoma, perianal, follicular differentiation, Hemorrhoid mimic

## Abstract

Perianal basal cell carcinoma (BCC) is a rare malignancy seldom encountered in clinical practice. It can clinically mimic benign anorectal conditions such as hemorrhoids or cysts, leading to diagnostic delay. We report the case of a 62-year-old Moroccan man presenting with a slowly enlarging perianal mass over four years, initially presumed to be hemorrhoidal. Histopathological analysis confirmed a diagnosis of BCC with follicular differentiation. Immunohistochemistry was positive for P63, CK5/6, and BCL2, and negative for melanocytic markers. Magnetic resonance imaging (MRI) showed no local invasion or distant metastasis. Wide local excision with tumor-free margins was performed, and the multidisciplinary team recommended surveillance. This case highlights the diagnostic challenge of perianal BCC mimicking benign lesions and emphasizes the need for histopathological confirmation. Follicular differentiation is exceptionally uncommon and should be distinguished from adnexal tumors such as trichoblastic carcinoma.

## Introduction

Anal cancer is uncommon, representing approximately 2.2% of all gastrointestinal malignancies. The predominant histologic type is squamous cell carcinoma (SCC), whereas perianal basal cell carcinoma (BCC) accounts for only about 0.2% of anorectal tumors [1]. Perianal BCC can mimic benign anorectal conditions such as hemorrhoids, ulcers, or cysts, often leading to delayed diagnosis and inappropriate initial management. Awareness of this possibility is crucial, as early biopsy of atypical or persistent perianal lesions enables timely diagnosis. Fewer than 200 cases of perianal BCC have been reported, including a recent meta-analysis [2]. Herein, we present a rare case of perianal BCC with follicular differentiation, which clinically mimicked hemorrhoids. The case highlights key diagnostic and management considerations and is discussed in the context of previously reported cases.

## Case presentation

**Figure 1:**
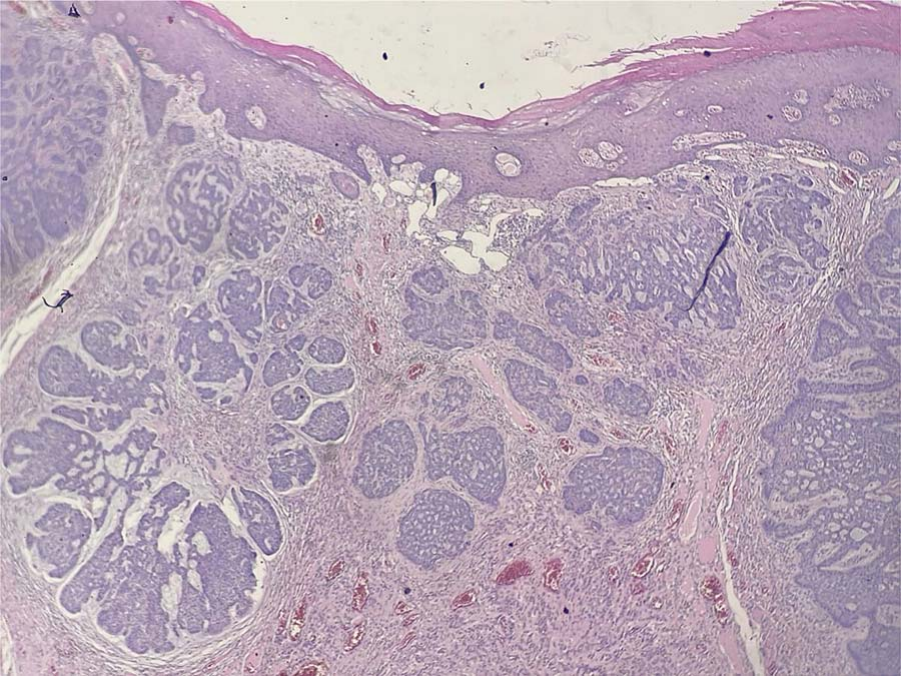
Carcinomatous proliferation arising from the epidermis and extending into the underlying dermis (HE x25).

**Figure 2:**
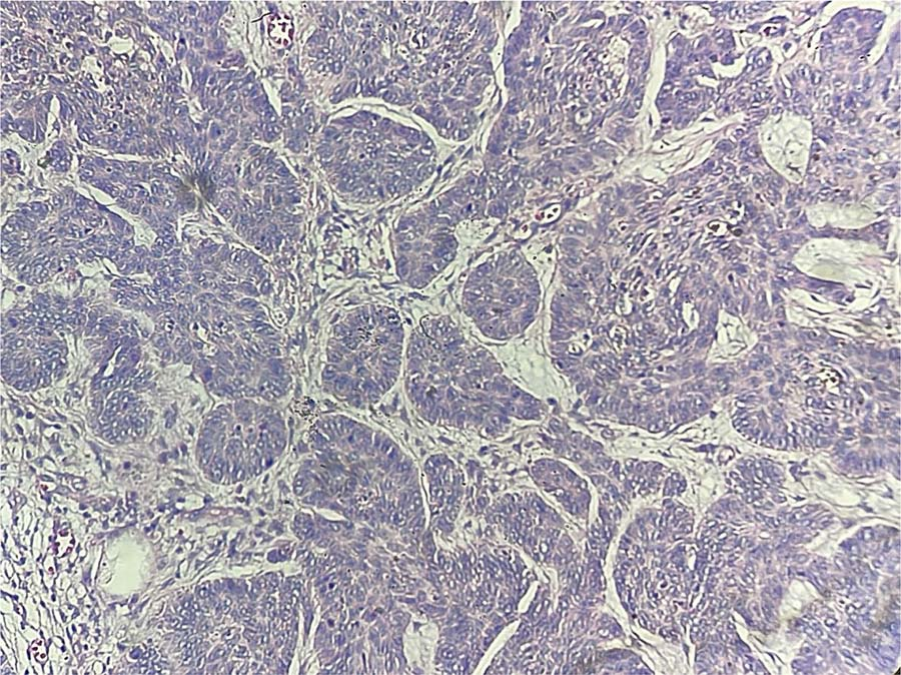
Nests and lobules, with small cystic spaces and follicular-like structures (HEx40).

A 62-year-old Moroccan man with no significant medical history presented with a slowly enlarging perianal mass evolving over four years. He reported mild anal discomfort during defecation but remained in good general condition. Physical examination revealed a fungating perianal lesion resembling external hemorrhoids. An excisional biopsy was performed. Histological examination demonstrated acanthotic squamous epithelium with a carcinomatous proliferation arising from the epidermis and extending into the underlying dermis (Fig. 1). The tumor measured 18 mm in greatest dimension and invaded 4 mm into the chorion. It was arranged in nests and lobules, containing small cystic spaces and follicular-like structures (Fig. 2). The basaloid cells exhibited peripheral palisading, retraction artifacts, vesicular nuclei with prominent nucleoli, and a mitotic index of 2 mitoses/10 high-power fields. The stroma was inflammatory, with numerous lymphocytes and plasma cells. No perineural invasion was noted. Surgical margins were clear: 10 mm from the superior and inferior edges, 5 mm from the internal, and 10 mm from the external and deep margins. Immunohistochemistry showed positivity for P63, CK5/6, and BCL2, and negativity for melanocytic markers, confirming the diagnosis of basal cell carcinoma with follicular differentiation. Pelvic MRI and thoracoabdominopelvic CT scan revealed no local invasion or distant metastasis. The tumor was staged as T1N0M0 (Stage I) according to the AJCC classification. The case was discussed in a multidisciplinary tumor board, and clinical surveillance was advised. No recurrence was observed after 12 months of follow-up.

## Discussion

Perianal BCC is an exceedingly rare variant of basal cell carcinoma and is often clinically mistaken for benign lesions such as cysts or hemorrhoids [1, 3]. This diagnostic overlap highlights the importance of early biopsy for any persistent or atypical perianal lesion. The most common anal malignancy is SCC, whereas perianal BCC is the least frequent among anorectal cancers [3]. Histologically, the basaloid variant of SCC may mimic BCC, but it typically arises within the anal canal and exhibits a more aggressive clinical course [4]. In contrast, BCC usually remains localized [5–7] and is effectively treated with wide local excision. SCC, however, requires chemoradiotherapy. Our case showed follicular differentiation an unusual histologic subtype that can resemble adnexal tumors such as trichoblastic carcinoma. Both entities share overlapping features, including basaloid cell morphology, lobulated architecture, and peripheral palisading. Trichoblastic carcinoma, however, often displays biphasic growth, deeper infiltration, high mitotic activity, and central necrosis [4]. Immunohistochemistry helps distinguish between these entities; markers such as BerEP4 and BCL2 are typically positive in BCC [8–10]. A recent meta-analysis found no single therapeutic modality superior to others regarding oncologic outcomes for perianal BCC [2]. Nevertheless, wide local excision with histologically clear margins remains the preferred approach. Radiotherapy may be considered for patients unfit for surgery. In conclusion, perianal BCC should be included in the differential diagnosis of chronic or atypical perianal lesions. Early histopathological evaluation is essential for accurate diagnosis and optimal management.

## Consent for publication

‘Written informed consent was obtained from the patient for publication of this case report and any accompanying images. A copy of the written consent is available for review by the Editor-in-Chief of this journal.’
